# Pneumothorax management: current state of practice in the UK

**DOI:** 10.1186/s12931-022-01943-9

**Published:** 2022-02-07

**Authors:** Rob J. Hallifax, Mark Roberts, Nicky Russell, Magda Laskawiec-Szkonter, Steve P. Walker, Nick A. Maskell, Najib M. Rahman

**Affiliations:** 1 Oxford Centre for Respiratory Medicine, Oxford University Hospitals NHS Trust, Oxford, UK; 2 Oxford Respiratory Trials Unit, University of Oxford, Churchill Hospital, Oxford, UK; 3 Sherwood Forest Hospitals NHS Foundation Trust, Sutton-in-Ashfield, UK; 4 Academic Respiratory Unit Bristol, University of Bristol, Bristol, UK; 5 NIHR Oxford Biomedical Research Centre, University of Oxford, Oxford, UK

**Keywords:** Pneumothorax, Management, Suction, Clamping, Air leak, Surgery

## Abstract

**Background and objective:**

Spontaneous pneumothorax is a common pathology but optimal initial treatment regime is not well defined. Treatment options including conservative management, needle aspiration (NA) or insertion of a small-bore chest drain. Recent large randomised controlled trials may change the treatment paradigm: comparing conservative and ambulatory management to standard care, but current guidelines need to be updated. The aim of this study was to assess the current “state of play” in the management of pneumothorax in the UK.

**Methods:**

Physicians and respiratory healthcare staff were invited to complete an online survey on the initial and subsequent management of pneumothorax.

**Results:**

This study is the first survey of pneumothorax practice across the UK, which highlights variation in practice: 50% would manage a large primary pneumothorax with minimal symptoms conservatively, compared to only 3% if there were significant symptoms; 64% use suction if the pneumothorax had not resolved after > 2 days, 15% always clamp the chest drain prior to removal; whereas 30% never do. NICE guidance recommends the use of digital suction but this has not translated into widespread usage: only 23% use digital suction to check for resolution of air leak).

**Conclusion:**

Whilst there has always been allowance for individual clinician preference in guidelines, there needs to be consensus on the optimum management strategy. The challenge the new guidelines face is to design a simple and pragmatic approach, using this new evidence base.

## Introduction

Spontaneous pneumothorax is a common pathology with an incidence of 17–24 and 1–6 per 100,000 population per annum for men and women, respectively [1, 2]. Primary Spontaneous Pneumothorax (PSP) conventionally refers to patients with no underlying lung disease; whereas those with lung conditions are termed Secondary Spontaneous Pneumothorax (SSP).

The most recent European Respiratory Society (ERS) [3] and British Thoracic Society (BTS) [4] guidelines for the management of pneumothorax were published in 2015 and 2010, respectively. The optimal initial treatment regime for PSP is not well defined: treatment options including conservative management, needle aspiration (NA) or insertion of a small-bore chest drain. Pleural drainage is recommended in patients with a large pneumothorax and/or symptoms initially with NA and then, if unsuccessful, chest drain insertion. However, two large randomised controlled trials (RCTs) have recently been published which may change the treatment paradigm: comparing conservative [5] and ambulatory management [6] to standard care, and the guidelines are due to be updated. The aim of this study was to assess the current “state of play” in the management of pneumothorax in the UK.

## Methods

Physicians and respiratory healthcare staff were invited, via personal email, mailshot from UK Pleural Society (www.pleura.org.uk) and advertisement on social media, to complete an online survey. The survey consisted of ten questions on the initial and subsequent management of pneumothorax. The questions were presented as multiple choice, but free-text answers were accepted. Data was collected on respondent’s job title, hospital type and main specialty.

## Results

The survey had 103 respondents. 71.6% were consultants. Fifty two (52.0%) of 100 respondents work in a district general hospital, and 48 (48.0%) from tertiary referral hospitals. 87 provided data on their main specialty: the majority 75 (86.2%) listed Respiratory Medicine as their main specialty; 7 (8%) were Emergency Medicine, 3 (3.4%) General Medicine, and one of each of acute medicine, and critical care. Hospitals from England, Scotland and Wales were all represented.

### Initial management

**Fig. 1:**
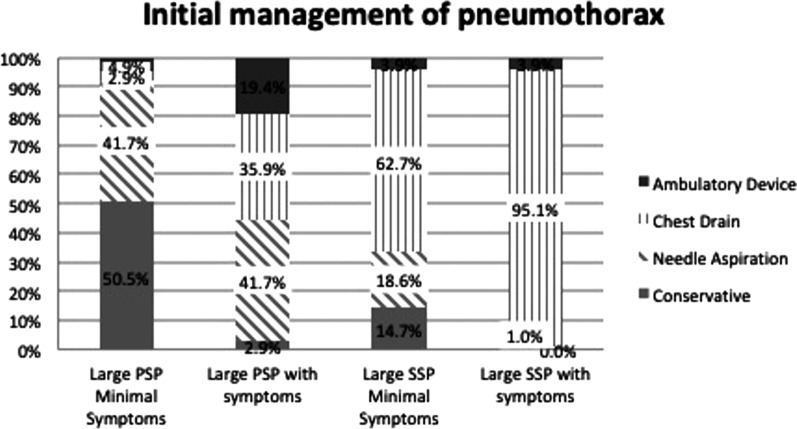
Choice of initial management by type of pneumothorax and symptoms. *PSP* Primary Spontaneous Pneumothorax, *SSP* Secondary Spontaneous Pneumothorax

**Fig. 2:**
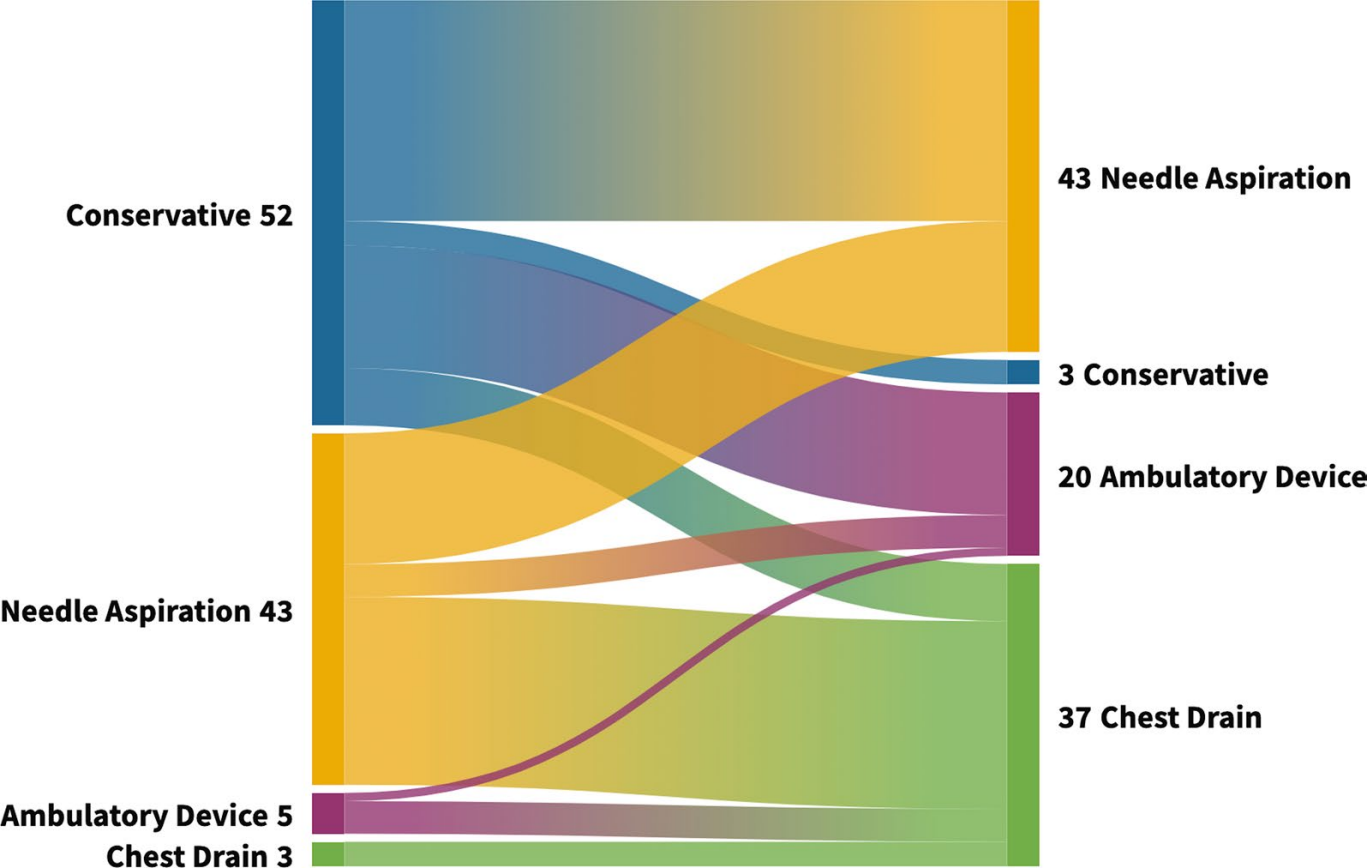
Sankey plot showing the flow of initial management choice for PSP from 1) Minimal symptoms (Left side) to 2) Ongoing symptoms (Right side)

Figure 1 shows the choice of initial management by type of pneumothorax (PSP or SSP) and symptoms. For patients with a large PSP and minimal symptoms, the majority (50.5%) would manage conservatively; although 41.7% opted to perform NA. If the patient had ongoing symptoms, only 2.9% manage a PSP conservatively; 41.7% would perform NA, 35.9% would insert a chest drain and 19.4% would insert an ambulatory device (see Fig. 2). For large SSP, the majority would insert a chest drain first for both patients with minimal (62.7%) and ongoing symptoms (95.1%) (Fig. 1).

Patients with Iatrogenic pneumothorax (IP), for example post-image guided lung biopsy, 54.0% would manage conservatively; although 23.0% stated their decision would be guided by size of pneumothorax, symptoms and/or progression on subsequent imaging.

### Ambulatory pathway

Twenty nine (28.2%) of 103 respondents stated that they currently have an ambulatory pathway to manage pneumothorax and a further 46 (44.7%) had plans to establish one in their hospital.

### Use of suction

64.1% (66/103) use suction if the lung is not re-expanded/ongoing air leak after 2 days, 10.7% (11) never use suction, 9.7% (10) use suction if the lung is not re-expanded/ongoing air leak after 4 days, 4.9% (5) use suction after referral for a surgical opinion, and the remaining 10% had other criteria (including non-resolution at 1 day, or if there were complications such as subcutaneous emphysema).

### Cessation of air leak

Once pneumothorax has resolved on CXR, cessation of air leak is assessed by “lack of bubbling”: on vigorous coughing by 35.3% (36/102) or with tidal breathing (13.7%, 14). 22.5% (23) of respondents use a digital suction device to assess for lack of air leak. 18.6% clamp the drain and repeat CXR. The remaining 10 (9.8%) respondents use a combination of the above options.

### Clamping

14.6% (15/103) of respondents always clamp the chest drain prior to removal; whereas 30.1% (31/103) never do. 55.3% (57/103) “sometimes” clamp under the following conditions: If the drain reinsertion would be difficult, for SSP, for those with prolonged air leak, if the patient had been on suction or history of early recurrence after previous drain removal.

### Post-removal Chest radiograph (CXR)

The majority (52.5%, 53/101) of respondents always repeat a CXR after chest drain removal, 18.8% (19/101) do not, and 28.7% (29/101) sometimes do (for example, if the drain reinsertion was deemed to be difficult, for all SSP, or if there was any uncertainty about on-going air leak).

## Discussion

This study is the first survey of pneumothorax practice across the UK. Despite an RCT in conservative management [5], only 51% of respondents would conservatively manage a patient with a large PSP with minimal symptoms and this reduces to 2.9% if the patient has ongoing symptoms. Similarly, only 19% would use an ambulatory device to manage a PSP with ongoing symptoms despite recent RCT data suggesting ambulatory management is effective [6]. There are a number of reasons has this evidence may not have influenced practice: clinicians may be unaware of the data, may not believe the results are valid or reflect their population, or may not feel empowered to act on the data due to lack of supporting facilities (e.g. ambulatory care unit). In addition, despite guidelines suggesting initial treatment with NA, only 36% in our survey would opt directly for chest drain insertion. This disparity with guidelines has been documented previously in case series and a previous survey of practice in 2000 [7].

Our survey highlights the variation in other controversial aspects of pneumothorax: detection of cessation of air leak, the use of “clamping” (obstructing the tube by turning off a 3-way tap or attaching a physical clamp to a drain), the use of suction, and whether to perform a post-removal CXR. NICE guidance [8] (based mainly on extrapolated data on surgical patients, and therefore possibly not applicable to this patient group) recommends the use of Thopaz + (digital suction) but this survey suggests that this has not translated into widespread usage: only 23% use digital suction. The remainder use a variety of criteria to assess for air leak: “bubbling” on underwater seal, or clamping. Historically, clamping has split clinicians into two camps: those in favour suggest that the rationale behind clamping is that a small air leak may not be easily detectable, but by blocking the chest drain for a few hours any leak would cause enlargement of the pneumothorax, and the drain can be unclamped easily (rather than necessitating further drain insertion); those against state that clamping any drain is dangerous as it risks tension pneumothorax.

## Conclusion

Whilst there has always been allowance for individual clinician preference in guidelines, there needs to be consensus on the optimum management strategy. The challenge the new guidelines face is to design a simple and pragmatic approach, using this new evidence base. It may be that a more personalised, stratified approach to treatment decision-making in PSP could minimise this variation, but as yet, there are no good predictors of early treatment failure. Failure of medical management (i.e. ongoing air leak at Day 4) may be predicted by digital airflow measurement [9] but whether this can be translated into a new clinical pathway needs to be confirmed in an RCT. Further data and the updated ERS and BTS guidelines are eagerly awaited. The real test, however, will be in the future education and implementation.

## Data Availability

Dr Hallifax is the guarantor of the data. Data will be made available to share with researchers on reasonable request.

## Abbreviations

BTS: British Thoracic Society
CXR: Chest radiograph
ERS: European Respiratory Society
IP: Iatrogenic Pneumothorax
NA: Needle Aspiration
NICE: National Institute for Clinical Excellence
PSP: Primary Spontaneous Pneumothorax
RCT: Randomised Controlled Trial
SSP: Secondary Spontaneous Pneumothorax
UK: United Kingdom
